# Plant Root Phenotyping Using Deep Conditional GANs and Binary Semantic Segmentation

**DOI:** 10.3390/s23010309

**Published:** 2022-12-28

**Authors:** Vaishnavi Thesma, Javad Mohammadpour Velni

**Affiliations:** 1 School of Electrical and Computer Engineering, University of Georgia, Athens, GA 30602, USA; 2 Department of Mechanical Engineering, Clemson University, Clemson, SC 29634, USA

**Keywords:** plant root phenotyping, deep learning, conditional generative adversarial networks, crop monitoring

## Abstract

This paper develops an approach to perform binary semantic segmentation on *Arabidopsis thaliana* root images for plant root phenotyping using a conditional generative adversarial network (cGAN) to address pixel-wise class imbalance. Specifically, we use Pix2PixHD, an image-to-image translation cGAN, to generate realistic and high resolution images of plant roots and annotations similar to the original dataset. Furthermore, we use our trained cGAN to triple the size of our original root dataset to reduce pixel-wise class imbalance. We then feed both the original and generated datasets into SegNet to semantically segment the root pixels from the background. Furthermore, we postprocess our segmentation results to close small, apparent gaps along the main and lateral roots. Lastly, we present a comparison of our binary semantic segmentation approach with the state-of-the-art in root segmentation. Our efforts demonstrate that cGAN can produce realistic and high resolution root images, reduce pixel-wise class imbalance, and our segmentation model yields high testing accuracy (of over 99%), low cross entropy error (of less than 2%), high Dice Score (of near 0.80), and low inference time for near real-time processing.

## 1. Introduction

Monitoring plant root morphology, also known as root phenotyping, is imperative to understanding a plant’s behavior in terms of nutrient absorption, growth, and response to environmental changes in soils [1,2]. Root phenotyping involves the characterization of a plant’s root system architecture (RSA) throughout plant growth such as root counting, thickness, length, and width. Roots help anchor plants above the ground and provide insight on a plant’s development and survival potential as variations in crop genotypes are developed, soil fertility changes, and efficient resource absorption becomes a priority to meet the rising food and crop demand. Therefore, root phenotyping allows for a comprehensive understanding of plant fitness under adverse conditions and for yield prediction [3,4].

Manual root phenotyping is very arduous as roots are usually small, thin, transparent, and most importantly, underground. Traditional root phenotyping had often been conducted by physically uprooting plants manually or by using unmanned ground vehicles (UGVs) for visual analysis. However, removing plants from the ground can easily damage roots. Furthermore, roots that are cored from the soil are later washed, which can result in root drying [5]. These damages on roots impede proper analysis of plant health. Thus, it is necessary to develop alternative, nondestructive, accurate, and robust analytic methods to automatically phenotype plant roots to monitor plant health [6].

Nondestructive crop monitoring typically utilizes a combination of traditional and modern computer vision methods, including pixel-based image processing, magnetic resonance imaging (MRI), X-ray tomography, 3D image construction, and machine and deep learning [3,4,7,8]. Moreover, these methods are popularly combined together to phenotype RSA automatically in both controlled and field environments. Plants grown in controlled environments have been placed in clear containers or gel media to easily and nondestructively examine RSA using imaging systems [4]. Specifically, segmenting roots from their background in 2D images using deep learning models has been popular to accurately and clearly visualize root health and temporal development, and to gain a comprehensive understanding of RSA [6]. Several works have addressed segmenting roots from their background using a combination of traditional and modern computer vision methods.

For example, the authors in [1] used pixel-based preprocessing on rice root images to discard background and maintain images with majority roots. Also, they used a sliding window approach to select smaller patches of these majority root images and fed them into two segmentation models. Furthermore, the authors in [2] developed a convolutional neural network (CNN) based off of SegNet, a popular segmentation network, to segment soybean roots from dense background soil. Similarly, the authors in [5] used U-Net, a classic segmentation model, to segment images of chicory roots growing in clear containers filled with soil. Similar to the efforts in [1,9], the authors in [3] compared various deep semantic segmentation models by performing traditional image transformations and randomly searching for patches with reduced pixel imbalance. They used their augmented dataset to train several segmentation models for comparison. Additionally, the authors in [7] used DeepLabv3+, another state-of-the-art deep encoder-decoder semantic segmentation model, for automatic segmentation of cotton roots. Their dataset was small and consisted of 10 training images that were sliced into patches. The authors in [10] used FutureGAN on images of *Arabidopsis thaliana* leaves and roots for growth and behavior prediction. Specifically, they train a generative model progressively beginning with low resolution images and adding additional layers for higher resolution images for detailed images [11]. Lastly, the authors in [12] used the same dataset as in [3] to perform a study on using various loss functions and parameters while training SegNet and U-Net for segmenting main and lateral roots from their background.

While several works have addressed root phenotyping via image segmentation, they rely heavily on traditional data augmentation methods to reduce pixel imbalance caused by the sparsity of roots in images themselves or only training small datasets. Traditional data augmentation techniques involve image transformations, color channel modifications, cropping, or patch creation. While these methods can drastically increase the size of the dataset, they require extensive storage, time, domain knowledge, and trial and error [13]. Also, these efforts may result in poor segmentation results such as model overfitting from oversampling the data, or reduced information learned from downsampling the data. Furthermore, these trained models are not generalizable to different datasets of root images that contain complete RSA instead of patches [14]. As such, additional pre- and post-processing would always be required for these models to create patches that reduce pixel imbalance (pre-processing) and interpret RSA development correctly (post-processing) [15]. Therefore, it is necessary for the segmentation models to learn from complete RSA data to provide accurate, generalizable results for root phenotyping quickly.

Modern generative methods, such as generative adversarial networks (GAN), have been employed for reducing class imbalances as they are able to create higher dimensional data without prior knowledge of the data distribution [13]. Thus, the generated data from GAN models aid to reduce class imbalance using specific oversampling techniques. For example, the authors in [16] used a conditional GAN to perform cassava root counting on both real and generated images. Specifically, a conditional GAN was used to generate more images with minority root classes to increase dataset size. Also, the authors in [17] used GAN to perform root restoration on *Arabidopsis thaliana* plants. The use of GAN for high resolution image generation allowed for missing root parts to be obtained for accurate analysis of root morphology and counting. Lastly, the authors in [10] used GAN also on *Arabidopsis thaliana* plants to forecast future root growth from young plant images.

The contribution of this work is in segmenting root images that contain complete RSA while reducing pixel-wise class imbalance. To achieve that, we use a high definition conditional GAN, Pix2PixHD, to generate realistic and high resolution images with complete RSA and their corresponding annotations to reduce the pixel-wise class imbalances between root and background of *Arabidopsis thaliana* root images. Furthermore, we use our generated dataset to perform binary semantic segmentation using SegNet. Our approach involves training two deep learning models to increase our dataset, reduce pixel-wise class imbalance, and perform semantic segmentation for the root phenotyping. This work aims to provide more generalizable segmentation results of plant root images that contain the complete RSA compared to current methods that use patches of root images, thereby increasing domain knowledge during learning.

The remaining sections of the paper are as follows: Section 2 provides a background to generative adversarial networks and its purpose for root phenotyping; Section 3 details our methodology for generating realistic images by training an image-to-image translation cGAN and training our binary semantic segmentation model; Section 4 shows our results and analysis of our experiments; Section 5 provides a discussion acknowledging current issues and future work; and Section 6 provides concluding remarks.

## 2. Background

Ideal class balance in datasets is present where there exists an even distribution for every class sample. However, realistic datasets do not always maintain perfect class balance and some classes may be more prevalent than others. It is also possible that non-desired classes are more prevalent than desired ones such as background. Datasets with class imbalance used for deep learning tasks, such as classification or segmentation, result in poor model performance.

In root phenotyping, it is common for root datasets to have class imbalance in terms of scarce amounts of roots in comparison to background, as roots are typically thin. This class imbalance is crucial to address when developing root phenotyping models. Traditional data augmentation techniques, such as cropping and patch creation, are not sufficient for improving segmentation tasks since these efforts do not adequately represent RSA and are expensive in terms of storage and time. However, generative models have shown prospect in reducing class imbalance even for semantic segmentation tasks [15].

Generative modeling is a type of unsupervised learning task that learns patterns from input data to create new samples similar to the input data. Generative adversarial networks (GANs), a type of generative modeling, contain two submodels: a generator model, *G*, and a discriminator model, *D* [18]. The generator is trained to create new samples similar to the input data and labels. The discriminator is simultaneously trained to classify if the input from the original dataset or the generator is real or not. The goal for the generator during training is to maximize the likelihood that the discriminator fails to determine the correct classification of the input data. This would indicate that the generator is creating plausible examples nearly indistinguishable from the original input data. Therefore, the relationship between these two models represents a two-player min-max game as

(1) $$\begin{array}{c} \min_{G}\max_{D}V\left(D,G\right)=E_{x}\left[\log D(x)\right]+\\ E_{z}\left[\log\left(1-D(G(z))\right)\right], \end{array}$$

where $E_{x}$ is the expected value over all samples in the dataset, log(D(x)) is the probability that the discriminator has determined that a sample is real, and $E_{z}$ is the expected value of the random input samples being fed into the generator. Ideally, the loss is minimized when both expected values are equivalent, indicating that the generator is creating nearly indistinguishable samples from the original data and the discriminator has a 50% chance of correctly determining if the sample is real or not.

A subset of GANs include conditional GANs (cGANs) where the input data being fed into the generator model is conditionally coupled with auxiliary metadata [19]. The coupled metadata may include a class label, numerical values, or images. The discriminator model is similarly conditioned where its input is now both the auxiliary metadata and original or generated data. This type of GAN allows for the generator to create data belonging to a particular domain. Similarly, cGANs also play a two-player min-max game as

(2) $$\begin{array}{c} \min_{G}\max_{D}V\left(D,G\right)=E_{x}\left[\log D(x|y)\right]+\\ E_{z}\left[\log\left(1-D(G(z|y))\right)\right], \end{array}$$

where *y* is the auxiliary metadata coupled with the input samples, log(D(x|y)) is the probability that the discriminator has determined that a sample is real given the concatenated conditional attribute *y*, and G(z|y) is the generator function for a sample *z* given the concatenated conditional attribute *y*.

The benefits of GANs are primarily their use for data augmentation by increasing the size and quality of an original dataset. Data augmentation usually increases the performance of models in terms of accuracy and generalizability. GANs, specifically, can also perform data augmentation by modelling higher dimensional data such as high resolution images, artwork, and image-to-image translation. cGANs for image-to-image translation are done by transforming an image from one domain to another while maintaining the content of the source image and modifying some visual attributes [20,21]. These types of cGANs must be trained to learn a mapping that can generate a new image similar to a target image while maintaining the content in the source image. In our work, we use an image-to-image translation cGAN to generate photorealistic and high resolution images of roots to reduce pixel-wise class imbalance in our root dataset. We utilize the benefits of cGAN to generate more root images with complete RSA by coupling the input root images with their annotations. This allows for the generated dataset to be similar to the original root dataset and contain complete RSA images.

## 3. Methodology

### 3.1. Dataset Acquisition

**Figure 1:**
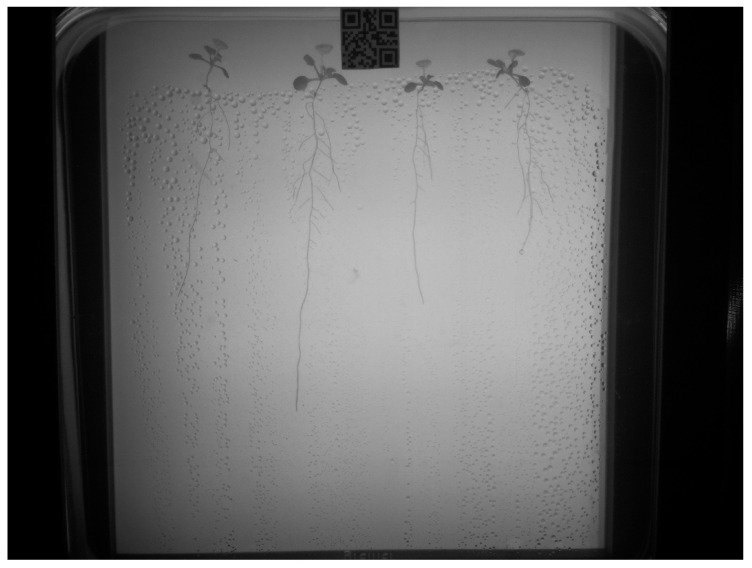
Example of a captured image frame of *Arabidopsis thaliana* plant growing in a controlled, indoor environment.

We use the dataset from the root segmentation challenge and the research conducted by the authors of [3]. The authors’ dataset consists of *Arabidopsis thaliana* plants growing in controlled, indoor environment inside clear gel Petri boxes. The growing periods varied between two and four weeks, and each Petri box contained four *Arabidopsis thaliana* plants. The authors used Raspberry Pi and four infrared cameras to capture RGB image frames of the plants’ roots growth over time in near-infrared lighting every twelve hours. An example of the growing conditions captured by the RGB camera used is seen in Figure 1. The resolution of each image is 3280 × 2464.

**Figure 2:**
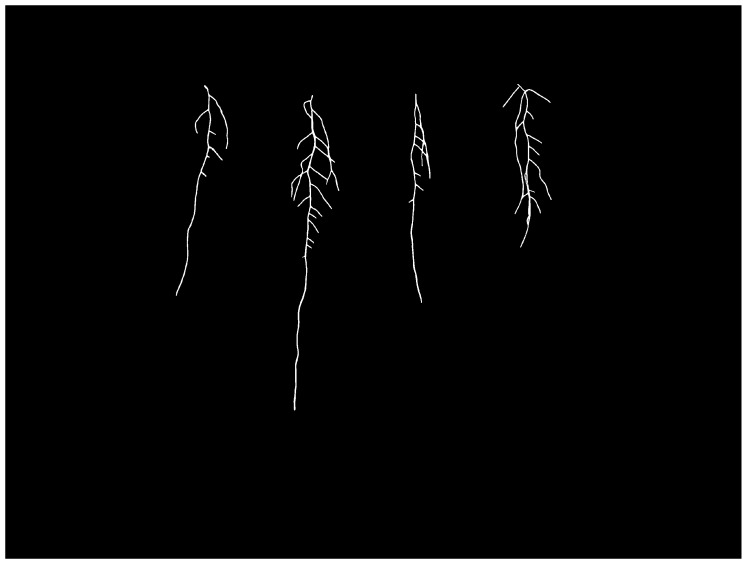
Example of the annotation corresponding to Figure 1.

A portion of the captured image frames were annotated for training segmentation models by the authors in [3]. In our experiments, we used 198 of the annotated images for binary segmentation. An example of the binary annotations that correspond to Figure 1 is shown in Figure 2.

The binary annotations were stored as MRI medical image format and were extracted using ITK-SNAP [22]. We use these 198 image frames and their corresponding binary annotations to feed into our cGAN and segmentation models for training. The average root to background pixel ratio of these annotations is 1:100, which indicates severe pixel-wise class imbalance for this dataset.

### 3.2. Semantic Map Creation

**Figure 3:**
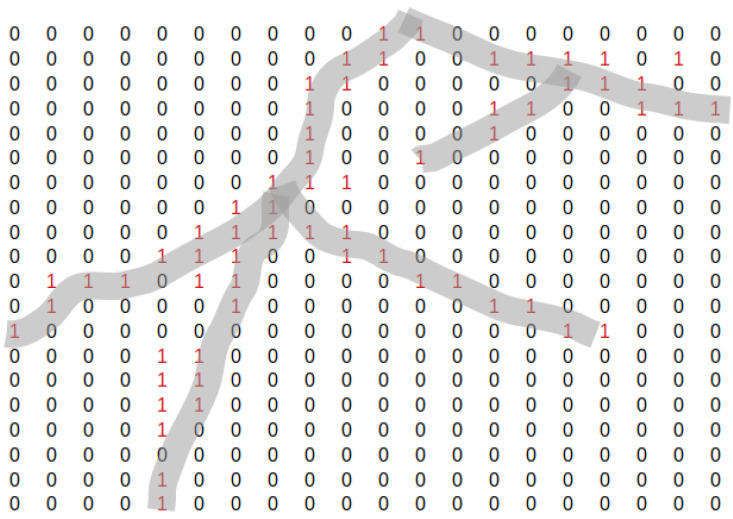
An illustrative example of semantic label map on a patch of a root image. The patch example is seen as a matrix, where indices with the value of 0 correspond to the background class and those with the red value of 1 correspond to the root class. Since the width of roots’ annotations is between 1 and 3 pixels, we highlight the red indices here in light gray so the form of the root is clearly visible.

To train both our cGAN and segmentation models, we converted the binary annotations of each image into semantic maps, where each pixel is labelled as belonging to a particular class from 0 to N−1, where *N* is the total number of classes. In our work, we have labelled two classes using Python Image Library (PIL), which include the background pixels as 0 and root pixels as 1. The semantic maps are created by first converting the RGB image annotations from 8-bit color to 8-bit gray-scale. Next, each white pixel corresponding to the roots in the gray-scale binary annotation is set to the value of 1. We store these semantic label maps for each annotation as a new image label file. An example of our semantic map creation is seen on a small patch of a root image in Figure 3.

### 3.3. cGAN Model Selection and Training

For our cGAN, we chose to use the Pix2PixHD architecture developed by [9] to generate new realistic images to augment our root dataset. This model is based off of Pix2Pix cGAN developed by [20], where the generator model learns to translate semantic label maps to realistic images and the discriminator model tries to distinguish the real images from the generated translated images [9]. Specifically, the Pix2Pix cGAN uses both the original image and its corresponding semantic label map as its auxiliary metadata for training. The model uses a U-Net architecture as the generator model and produces low resolution images. For our experiments, we require high resolution images since the roots are thin and sparse with respect to the background.

**Figure 4:**
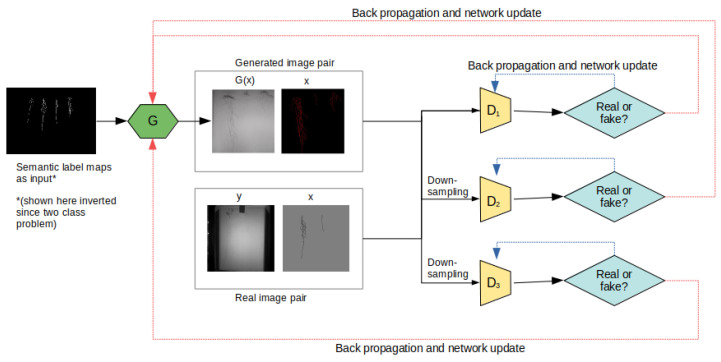
Pix2PixHD architecture.

Pix2PixHD builds on Pix2Pix by improving photorealism and resolution [9]. Specifically, Pix2PixHD incorporates a coarse-to-fine generator model, a multi-scale discriminator, and a robust adversarial loss function. The coarse-to-fine generator contains two subnetworks that are jointly trained on high resolution images. The multi-scale discriminator contains three discriminator models that are trained on different image sizes by downsampling its input images. The motive for the multi-scale discriminator is to reduce computational complexity of using one discriminator on high resolution images. Lastly, the robust adversarial loss stabilizes the generator during training. The architecture of Pix2PixHD used for this work is inspired from the one in [23] and is shown in Figure 4.

We use 163 out of 198 images from our dataset to train the Pix2PixHD model from scratch for 118 epochs. Specifically, we use Google Colaboratory to accommodate the high GPU memory requirement of Pix2PixHD. During each epoch, our model randomly cropped each image to reduce the amount of empty background and increase the ratio of root to background pixels. We set the batch size to 1 to reduce training time, the learning rate to 0.0002, and used the Adam optimizer. Lastly, we used two discriminators during training. Our model took approximately ten hours to train.

We use our trained cGAN to generate an additional 396 images using the remaining 35 images from our training dataset and by vertically flipping our original training data to be different than the data used to train the cGAN. Similar to the cGAN training procedure, the trained cGAN randomly cropped and flipped each image to generate the fake images and corresponding labels. Thus, we increased our original dataset by three folds and it finally consisted of 594 images and their corresponding annotations. Our Pix2PixHD codes are inspired from the GitHub repository https://github.com/NVIDIA/pix2pixHD, which were accessed on 1 April 2022.

Lastly, we processed the images and annotation from our generated dataset and the original dataset to be fed into our SegNet model for semantic segmentation. We resized both our datasets and their corresponding labels to 480 by 360 using PIL, as this is the required input size for training SegNet, and we converted the generated fake labels to segmentation maps using the same methodology discussed in Section 3.2.

### 3.4. Semantic Segmentation Model Selection and Training

**Figure 5:**
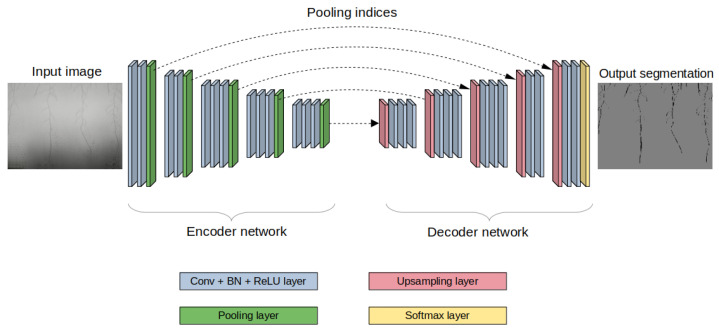
SegNet architecture.

For our semantic segmentation model, we chose SegNet, a popular state-of-the-art semantic segmentation model that is primarily used for road scene understanding and dense pixel-wise classifications [24]. Similar to various U-Net series, SegNet has an encoder-decoder architecture [25]. The encoder network is identical to the popular VGG16 convolutional layers, but the fully connected layers are removed to make the SegNet encoder part smaller and easier to train end-to-end. The encoder network contains encoder blocks that downsample the inputted RGB images using convolutional and max pooling layers. The decoder network contains decoder blocks that upsample the extracted features from the convolutions and finally apply pixel-wise classification. The output of each pooling layer from the encoder network is concatenated with an upsampling layer in the decoder network. Thus, there is 1 decoder block for every 1 encoder block. The architecture of SegNet used for this work is shown in Figure 5.

We feed the processed generated dataset, the original dataset, and both their corresponding annotations into our SegNet model to train from scratch. We train our model for 50,000 iterations and set the batch size to 5 to reduce the training time, the momentum to 0.9, the learning rate as 0.0001 and use the Adam optimizer again to automatically adjust the learning rate. Our SegNet codes are inspired from the GitHub repository https://github.com/aizawan/segnet, which were accessed on 1 November 2021.

### 3.5. Segmentation Postprocessing

Our segmentation results from our trained SegNet model show some gaps along the main root and lateral roots. To address this issue, we manually post-processed the segmentation results to close the gaps between main root and lateral roots. We chose a small image patch from our segmentation results and manually searched for gaps by converting the results to binary matrix similar to Figure 3, saved the matrix to a CSV file, and recorded the coordinates of the gaps’ endpoints. We defined each branch and main root as arrays of endpoints’ coordinates and connected the gaps between the endpoints by drawing white lines using PIL.

### 3.6. Evaluation Metrics

We use different evaluation metrics to gauge the performances of both our trained Pix2PixHD cGAN and SegNet segmentation models. For our cGAN model, we examine the performance based on the objective loss function given in (2) and the visual clarity of the generated images during training.

For our semantic segmentation model, we examine its performance using four metrics. The first is the cross entropy loss function at the end of training; the second is the overall accuracy of the model also measured at the end of the training; the third is the mean intersection-over-union (IOU) from testing our trained model on our testing set; and the last is the Dice Score also measured from testing our trained model on our testing set. Cross entropy is another popular loss function used to evaluate the performance of deep learning models. This loss function determines the difference between two probability distributions for a random variable or event [26]. For segmentation tasks, cross entropy loss aims to minimize pixel-wise error, especially in high class imbalance scenarios as in our experiments. Specifically, cross entropy loss is defined as

(3) $$L_{CE}\left(y,\hat{y}\right)=-\left(y\log\hat{y}+\left(1-y\right)\log\left(1-\hat{y}\right)\right),$$

where $y,\hat{y}\in {\left\{0,1\right\}}^{N}$ and *y* is the true class label and $\hat{y}$ is the predicted class label. Ideally, the value of (3) should be near 0 for a well performing model.

The accuracy metric is a global average of pixels being correctly classified as being root or background. Each pixel can be classified as true positive (TP), false positive (FP), true negative (TN), or false negative (FN) as described in [27]. Ideally, the value of accuracy should be near 1 for a well performing model.

Furthermore, mean IOU is another common evaluation metric to determine the overall performance of a trained semantic segmentation model. Specifically, mean IOU determines the percent overlap of the ground truth and the trained model’s prediction. Based on the aforementioned possible pixel classifications, mean IOU can be defined as

(4) $$IOU=\frac{TP}{TP+FP+TN}.$$

Ideally, the value of mean IOU should be near 100% for perfect segmentation overlap. However, achieving this is very difficult for root segmentation tasks as roots are very sparse and as thin as 1 pixel in width.

Dice Score is another metric used for evaluating the accuracy of the segmentation models; it is similar to F1 Score but used for segmentation tasks [28]. Dice Score can be defined as a function of mean IOU as

(5) $$Dice=\frac{2*IOU}{1+IOU}.$$

Ideally, the value of Dice Score should be near 1 for a well performing segmentation model.

Lastly, we compute the average inference time for our trained SegNet model to perform semantic segmentation in the testing dataset.

### 3.7. Model Comparison

Comparing our segmentation model’s performance with another group’s on the same dataset is warranted to establish a performance baseline. As such, we compare our results with the models developed and trained by [3], as they initially created the dataset used in this work. Specifically, we compare our segmentation results with their trained UNet model and their Deeply Supervised ResUNet (DSResUNet) model. The model weights were already trained on the same dataset for binary semantic segmentation and were provided on GitHub. Furthermore, it is important to note that the authors in [3] used traditional data augmentation techniques, such as Gaussian filtering and blurring, flipping, and patch creation, to reduce pixel-wise class imbalance.

The UNet model is similar to SegNet in that it uses an encoder-decoder architecture and produces a pixel-wise dense segmentation map. The authors in [3] modified the architecture of the UNet model by reducing the number of feature maps per convolutional layer, using average pooling, and using an exponential linear unit (ELU) activation function. The lighter version of UNet from these modifications allowed for the authors in [3] to have improved results beyond the UNet base model. Thus, we compare the performance of this model on our testing dataset with our trained SegNet model.

Contrastingly, the DSResUNet model uses ResUNet, another popular segmentation model, as a baseline by combining residual connections and deep supervision. Deep supervision involves incorporating additional loss terms in the intermediate convolutional layers. Furthermore, the original output of ResUNet is concatenated with two additional convolutional layers. The additional CNN layers refines the ResUNet results and the loss functions from each subnetwork are added and used for finetuning the DSResUNet model during training. The combination of these features allowed for the authors in [3] to have improved results beyond the ResUNet base model. As such, we also compare the performance of this model on our testing dataset with our trained SegNet model.

In our comparison, we feed our testing dataset into both the pretrained DSResUNet and UNet models for binary semantic segmentation. The segmentation results from both the DSResUNet and UNet model are evaluated using the same performance metrics, except for the cross entropy loss, as described in Section 3.6.

### 3.8. Summary of Methodology

**Figure 6:**
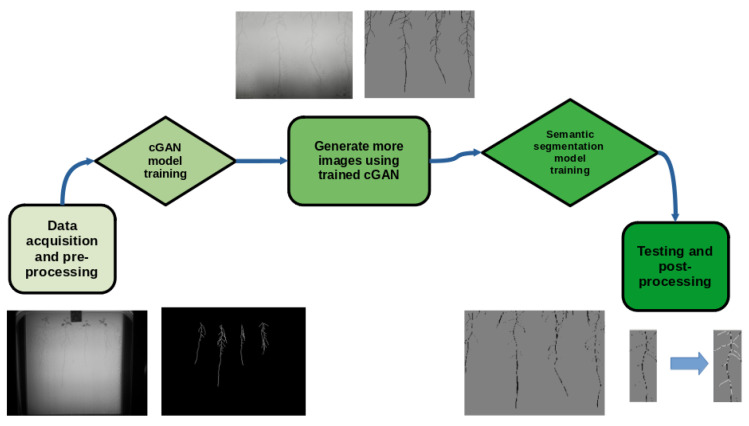
Summary of our methodology in this work.

In Figure 6, we portray our pipeline including data acquisition and preprocessing, model training, and final result postprocessing as a clarified summary. The codes used in these experiments will be made available on our lab website.

## 4. Experimental Results

In this section, we provide results and discussions on the proposed root segmentation approach.

### 4.1. Pix2PixHD Results

**Figure 7:**
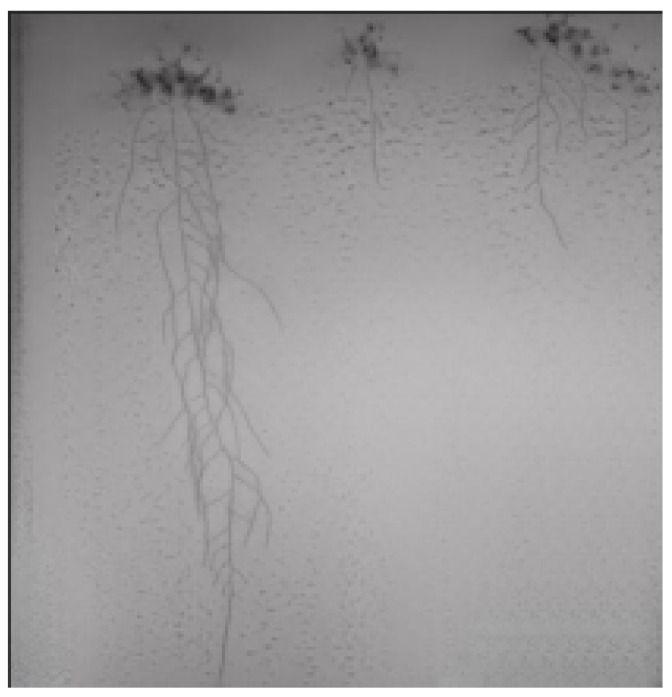
Example of a generated image from our trained cGAN. The roots are clearly visible and look similar to our original dataset. The *Arabidopsis thaliana* leaves are not translated in the generated images since semantic label maps were not created for them.

**Figure 8:**
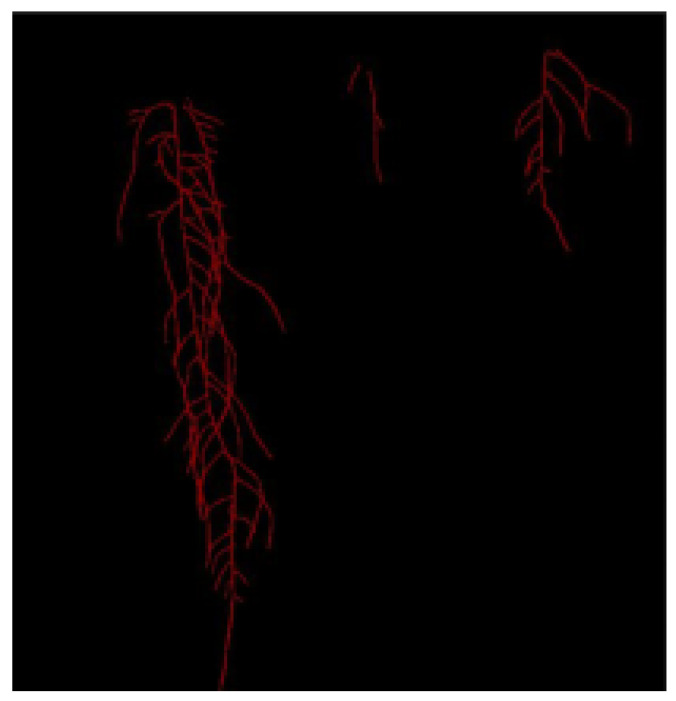
Example of the corresponding annotation from Figure 7. The annotations are clear and include the same generated root architecture present in Figure 7.

Using our trained Pix2PixHD cGAN, we generated an additional 396 images to augment our original 198 root image dataset. An example of a generated image and its corresponding annotation is shown in Figure 7 and Figure 8, respectively. Our generated images show that the *Arabidopsis thaliana* roots are similar to our original dataset as seen in Figure 1. Additionally, since our original annotations only contain annotated root pixels and considered the leaves as background, the generated images do not perfectly translate the *Arabidopsis thaliana* leaves. This is acceptable as we are mainly interested in generating photorealistic root images similar to our original dataset and in semantically segmenting the RSA from the background.

### 4.2. SegNet Results

We tested our trained SegNet model on 30 images comprised both of generated images from our trained cGAN and our original dataset. The performance of our model based on the aforementioned evaluation metrics discussed in Section 3.6 is given in Table 1. Our trained model shows high global average accuracy and Dice Score. Specifically, most pixels were correctly classified as either root or background. Additionally, the cross entropy loss of our trained model is very low and near zero. While the mean IOU and Dice Scores are not close to their ideal values, they are both still high given that our task involves segmenting thin roots and a portion of the testing dataset contains the original root images that contain a high pixel-wise class imbalance of 1:100. Lastly, the average inference time to process each image is small, and hence the method can be used for near real-time applications.

**Figure 9:**
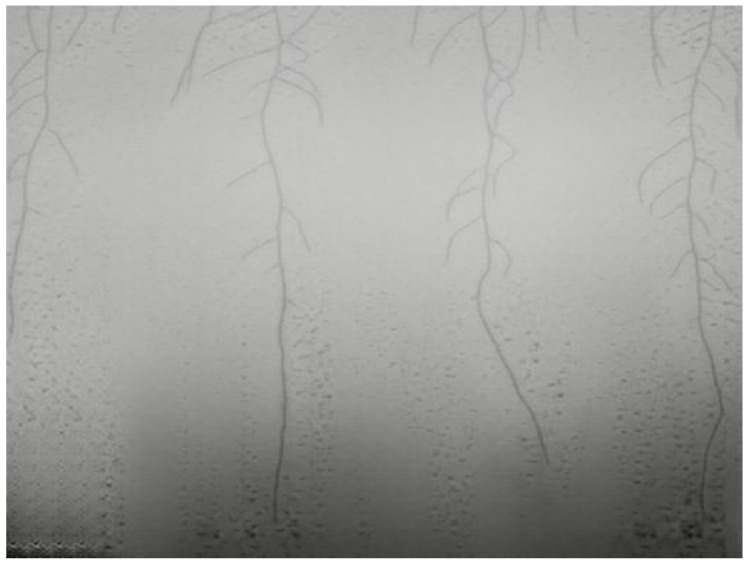
Corresponding original generated image as Figure 11. This image is used as example to show segmentation results of our trained SegNet model.

**Figure 10:**
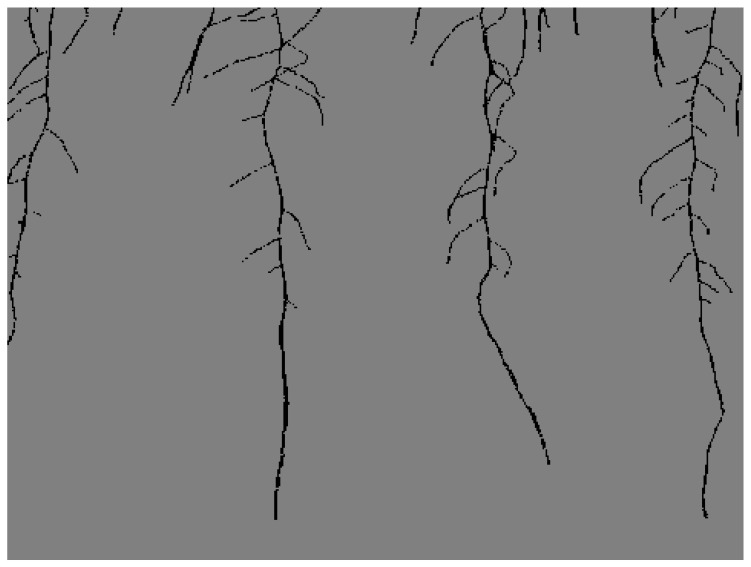
Corresponding annotation of Figure 9. This image is used as example to show segmentation results of our trained SegNet model.

**Figure 11:**
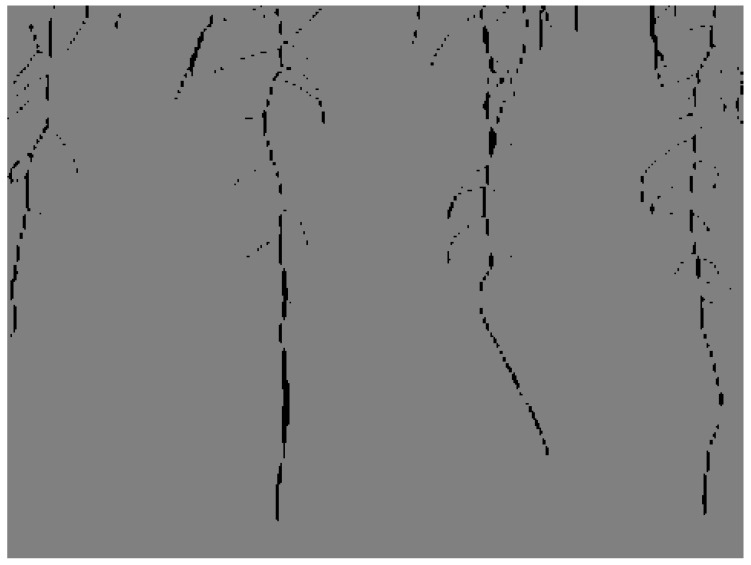
Example of semantic segmentation results from our trained SegNet model. The main and lateral root architectures are successfully segmented, but there are small gaps along them.

An example of our segmentation results is seen in Figure 9, Figure 10 and Figure 11. The image in Figure 9 is one of the generated images from our trained Pix2PixHD cGAN representing the *Arabidopsis thaliana* roots growing in the gel Petri dish. The corresponding annotation for the generated image is seen in Figure 10. The segmentation results of Figure 9 after feeding into our trained SegNet model is seen in Figure 11.

Our segmentation results demonstrate that most of the root architectures can be successfully segmented from the background using our trained model. However, there are visible gaps along the lateral and main roots. The lengths of these gaps are typically less than 10 pixels and thus can be mitigated using traditional pixel-based postprocessing to close the gaps as discussed in Section 3.5.

### 4.3. Postprocessing Results

**Figure 12:**
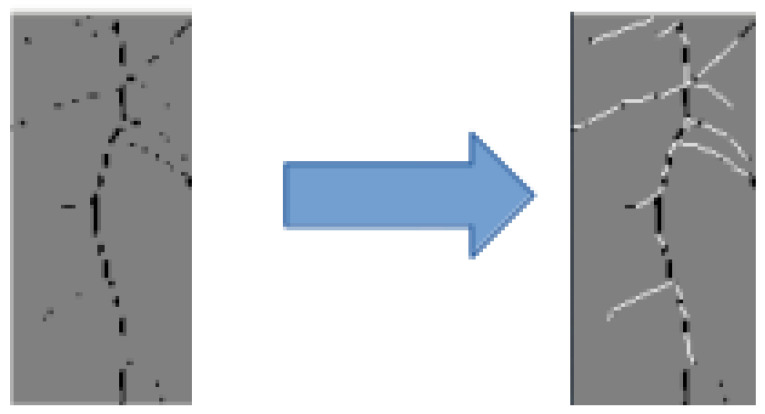
Example of postprocessing a patch of our segmentation results from Figure 11 using PIL. The gaps along the segmented lateral and main roots are closed using white lines.

From Figure 11, we select a small patch from the second root system from the left for closing the gaps along the lateral and main roots using pixel-based postprocessing. The selected patch is seen in left-hand subimage in Figure 12. We manually process this image and connect the gaps using thin white lines as shown in the right-hand subimage of Figure 12. Thus, our postprocessing method can effectively close small gaps from our segmentation results.

### 4.4. Model Comparison Results

The comparison between our trained SegNet model and both the DSResUNet and UNet models from [3] is shown in Table 2. Specifically, we compare our trained SegNet performance in terms of average accuracy, mean IOU, Dice Score, and inference time with the other two models.

It is evident from Table 2 that the global average accuracy of all three models is very high and above 99%. However, our trained SegNet models show significantly higher mean IOU and Dice Score metrics in comparison to both the DSResUNet and UNet models. This indicates that our trained SegNet model has superior performance in terms of accurately segmenting the RSA pixels from the background successfully. Lastly, the inference time for the pre-trained UNet model is superior to both the SegNet and DSResUNet models, likely due to its light-weight architecture.

## 5. Future Work

Firstly, additional work is needed for segmenting both lateral and main roots separately. This way, we can analyze RSA depth and width in relation to the crop health over time. To accomplish this, it is imperative that we use annotations that differentiate both the main and lateral roots. Specifically, the segmentation maps must be annotated with three classes instead of two: (0) background, (1) main root, and (2) lateral roots. Furthermore, pixel-wise class imbalance for both main and lateral roots will still be prevalent and must be addressed for both of these classes. Thus, we must generate more high resolution root images and annotations using our trained cGAN to reduce pixel-wise class imbalance. Lastly, using a state-of-the-art multi-class segmentation model, like UNet or SegNet will be necessary to segment both lateral and main roots from the original and generated datasets.

Secondly, it is noted that the dataset used in this research involved *Arabidopsis thaliana* plants grown in controlled environments with no access to soil nutrients [29]. This indicates that the plants grown lacked root interaction with soil nutrients and microbes, which potentially limited variations in growth and reduced nutrient availability. Thus, our trained SegNet model will be subject to further performance analysis to determine how well the RSA can be accurately segmented while in soil.

Lastly, manual gap closing efforts are timely and tedious. Thus, implementing automatic gap closing methods is important to increase segmentation accuracy for both mean IOU and Dice Score. Image inpainting may be a potential solution to automate manual gap closing present in segmentation results, as it is a deep learning method for reconstructing missing areas in images. This method will require extensive model training and tuning to ensure effective and automatic gap closing in segmented root images.

## 6. Concluding Remarks

In this paper, we present an approach to segment *Arabidopsis thaliana* root images from the background using a high resolution cGAN to reduce pixel-wise class imbalance and increase the size of our dataset. The results show that our trained Pix2PixHD cGAN model can generate photorealistic images of full root architectures with their corresponding annotations. Furthermore, our trained SegNet model can segment the RSA from both the original and generated datasets. The contribution of our work is that our trained models can effectively reduce pixel-wise class imbalance without the need for creating smaller patches, as patches do not sufficiently represent RSA. Thus, our experimental results demonstrate potentials in being generalizable to a variety of root images being fed into our model for segmentation.

## Figures and Tables

**Table 1:** Evaluation metrics for our trained SegNet model.

Metric	SegNet Performance
Cross Entropy Loss	0.020
Accuracy	0.991
Mean IOU	65.87%
Dice Score	0.7942
Inference Time	0.3002 s

**Table 2:** Evaluation metrics for our trained Chronoroot model comparisons with our trained SegNet model.

Metric	SegNet (Ours)	DSResUNet	UNet
Accuracy	0.991	0.994	0.995
Mean IOU	65.87%	42.78%	54.34%
Dice Score	0.7942	0.5582	0.6839
Inference Time	0.3002 s	0.2881 s	0.1728 s

## Data Availability

Not applicable.

## Abbreviations

The following abbreviations are used in this manuscript:

GAN	Generative Adversarial Network
cGAN	Conditional Generative Adversarial Network
UGV	Unmanned Ground Vehicle
MRI	Magnetic Resonance Imaging
CNN	Convolutional Neural Networks
RSA	Root System Architecture
PIL	Python Image Library
IOU	Intersection Over Union
TP	True Positive
FP	False Positive
TN	True Negative
FN	False Negative
ELU	Exponential Linear Unit
ResUNet	Residual UNet
DSResUNet	Deeply Supervised Residual UNet
